# Volatile Profiles of Sweet Breads Enriched with Orange and Lime Peel Flours

**DOI:** 10.3390/foods15213931

**Published:** 2026-11-02

**Authors:** Katheryn L. Vasquez-Gomez, Diner Mori-Mestanza, Lucas D. Muñoz-Astecker, Erick A. Auquiñivin-Silva, Dorila E. Grandez-Yoplac, Grobert A. Guadalupe

**Affiliations:** 1 Escuela de Posgrado, Programa de Maestría en Ciencia y Tecnología de los Alimentos, Facultad de Ingeniería y Ciencias Agrarias, Universidad Nacional Toribio Rodríguez de Mendoza de Amazonas, Higos Urco 342, Chachapoyas 01001, Peru; 73873969m1@untrm.edu.pe; 2 Escuela de Posgrado, Programa Doctoral en Ciencias para el Desarrollo Sustentable, Facultad de Ingeniería Zootecnista, Biotecnología, Agronegocios y Ciencia de Datos, Universidad Nacional Toribio Rodríguez de Mendoza de Amazonas, Chachapoyas 01001, Peru; 3 Grupo de Investigación en Innovación y Desarrollo Agroindustrial (GIIDA), Instituto de Investigación, Innovación y Desarrollo para el Sector Agrario y Agroindustrial (IIDAA), Facultad de Ingeniería y Ciencias Agrarias (FICA), Universidad Nacional Toribio Rodríguez de Mendoza de Amazonas, Higos Urco 342, Chachapoyas 01001, Peruerick.auquinivin@untrm.edu.pe (E.A.A.-S.); 4 Grupo de Investigación en Seguridad Alimentaria (GISA), Instituto de Investigación, Innovación y Desarrollo para el Sector Agrario y Agroindustrial (IIDAA), Facultad de Ingeniería y Ciencias Agrarias (FICA), Universidad Nacional Toribio Rodríguez de Mendoza de Amazonas, Higos Urco 342, Chachapoyas 01001, Peru

**Keywords:** volatile compounds, food composition, peel flours, by-products of citrus

## Abstract

This study characterized the volatile organic compounds of sweet breads formulated with 2% and 4% orange (*Citrus sinensis*) (C.S.) and lime (*Citrus limetta* Risso) (C.L.) peel flours by integrating gas chromatography–mass spectrometry (GC-MS) and multivariate analysis. A total of 165 volatile compounds belonging to 11 chemical families were identified. Citrus peel flour significantly modified the volatile profile (*p* ≤ 0.05), primarily reducing furans, carboxylic acids, aldehydes, ketones, and pyrazines while increasing terpenes, aldehydes, alcohols, and esters. Monoterpenes reached their highest relative abundance in C.L. 2% (45.029 ± 2.284%), whereas sesquiterpenes progressively increased to 6.183 ± 0.762% in C.L. 4%. Several volatile compounds were tentatively identified in the formulations enriched with citrus peel flours. Limonene exhibited the highest relative abundance (14.957 ± 1.406%) in the C.L. 2% formulation, followed by the aldehydes neral (0.258 ± 0.041%) and citral (0.592 ± 0.102%), both detected in the C.L. 4% formulation. Other identified compounds included terpene alcohols, such as geraniol, citronellol, linalool, terpinen-4-ol, and α-terpineol, as well as esters, including geranyl acetate, (Z)-3,7-dimethylocta-2,6-dien-1-yl acetate, and α-terpinyl acetate. These findings provide scientific evidence regarding changes in the profile of volatile compounds associated with the incorporation of citrus peel into sweet breads and support its practical application for diversifying the characteristics of sweet breads while promoting the valorization of agroindustrial byproducts.

## 1. Introduction

In recent years, growing interest in healthier and more nutritious foods has driven the development of new baked goods made with alternative ingredients that provide health-promoting compounds [1,2]. However, the inclusion of these ingredients also raises the need to understand how their chemical composition can influence the characteristics of these products [3].

Wheat flour is the most commonly used ingredient in breadmaking due to the technological properties of gluten; however, its substitution with other flours has been extensively studied to improve its nutritional and functional value. These blended flours can provide higher amounts of fiber, lipids, antioxidants, and other health-promoting compounds [4,5]. In a previous study, we evaluated the risk-benefit ratio of incorporating *Citrus sinensis* (orange) and *Citrus limetta* Risso (lime) peel flours into sweet breads at inclusion levels of 2% and 4%. The results showed that these formulations exhibited adequate technological feasibility and provided significant nutritional and functional benefits; furthermore, it was determined that the risk values were less than 1, even at a 4% inclusion level, indicating a non-significant toxic risk and supporting the safety and feasibility of these formulations for further studies in baked goods [6].

The aroma of bread is associated with a complex mixture of volatile organic compounds (VOCs) from different chemical families, with alcohols being the most abundant, followed by aldehydes, ketones, esters, acids, hydrocarbons, pyrazines, furans, and sulfur-containing compounds [7,8]. More than 300 to 400 volatile molecules have been identified in bread, such as (E)-2-nonanal (green, tallow), 3-methyl-1-butanol (balsamic, alcoholic), hexanal (green), and 2,3-butanedione [7,9]. These compounds are formed primarily by the metabolic activity of yeast and lactic acid bacteria, by the oxidation of lipids in the flour during mixing, and by Maillard reactions on the dough surface [10,11].

The composition of volatile compounds in baked goods can vary with the ingredients used and the chemical changes that occur during processing [7,12]. Several studies have used gas chromatography coupled with mass spectrometry (GC-MS) to characterize these compounds in flours and baked goods made with novel plant-based food ingredients [13,14]. Furthermore, the characterization of flours made from the peels of various citrus fruits has revealed the chemical diversity of these raw materials, primarily due to terpenes such as limonene, β-myrcene, linalool, α-pinene, and valencene, highlighting their potential for various food applications [15]. However, it remains unclear how citrus species and incorporation level influence the volatile profile of baked goods. Therefore, this study offers a novel perspective by comparing the volatile compound profiles of sweet breads made with C.S. and C.L. peel flours, incorporated at 2% and 4%. This approach allows analysis of both differences among citrus species and variations in incorporation level, contributing to an understanding of how these raw materials affect the volatile composition of the sweet bread matrix.

## 2. Materials and Methods

### 2.1. Production of Citrus Flours

Citrus fruits *C. sinensis* (C.S.) and *C. limetta* Risso (C.L.) were harvested when ripe from orchards located in the Magdalena district, Chachapoyas province, Amazonas region, Peru. The fruits were then transported to the Engineering Laboratory at the Toribio Rodríguez de Mendoza National University of Amazonas, where they were disinfected with 0.1% sodium hypochlorite. The peels were manually separated from the remaining parts and cut into pieces measuring approximately 1 cm^2^. The peels were weighed and placed on stainless steel trays for drying in a convection tray dryer (GUNT AMBURG, CE130, G.U.N.T. Gerätebau GmbH, Barsbüttel, Germany) at 60 °C until they reached a constant weight (approximately 6–7 h). Once the samples were dehydrated, the grinding process began, followed by sieving (0.5 mm sieve). The flours were vacuum-packed and stored at −20 °C until analysis. The physicochemical characterization of the flours was performed previously and is reported in our earlier study [6], which describes the main physicochemical indicators of both flours.

### 2.2. Formulation of Sweet Breads

The sweet breads made by C.S. and C.L. with 2% and 4% (representing the substitution of the wheat flour fraction) were produced mechanically, according to the formulation in Table 1. First, the sugar was dissolved in water for 2 min. Next, the remaining ingredients (wheat flour/citrus peel flour, sugar, vegetable shortening, freeze-dried dry yeast, and eggs) were added to the mixture and kneaded for 5 min to obtain a homogeneous dough. The dough was then rounded and shaped. Once the dough was shaped, it was left to proof for 2 h at 27 °C. Next, the baking stage began at 180 °C for 15 min in a rotary oven [6]. The amount of water added was kept constant across all formulations to maintain consistent processing conditions and to evaluate the effect of the substitution level on product characteristics. The ingredients are expressed per 100 g of total formulation.

### 2.3. Volatile Compounds

The volatile compounds (VOCs) in sweet bread were extracted using headspace solid-phase microextraction (HS-SPME) with a divinylbenzene/carboxene/poly (dimethylsiloxane) (DVB/CAR/PDMS) fiber 50/30 μm thick (Supelco, Sigma-Aldrich NV, Bornem, Belgium) and analyzed using a gas chromatography–mass spectrometry system (7890B GC System and 5977B MSD, Agilent Technologies^®^, Santa Clara, CA, USA), following the method described by Si et al. [16] and Mori-Mestanza et al. [17] with modifications. Two grams of sample were used; these were transferred to a 20 mL vial and subsequently sealed hermetically with an aluminum cap and a PTFE/silicone septum. For each formulation, three analytical replicates obtained from the same batch of sweet bread were analyzed. The samples were conditioned for 15 min at 60 °C in a digital heater (VWR, model No. 949VWHB2EUA), followed by 40 min of fiber exposure inside the vial at the same temperature to allow for the adsorption of volatile compounds. The fiber was then inserted into the manual injection port of the GC-MS system, which involved thermal desorption of the compounds extracted by the fiber (250 °C for 5 min).

GC conditions: DB-5MS UI capillary column (60 m × 250 µm × 1 µm) with helium as the carrier gas (flow rate: 1 mL/min). The oven temperature program was set as follows: the column temperature was initially maintained at 40 °C for 4 min, then increased at 6 °C/min to 230 °C for 12 min. Injection was performed in splitless mode.

Mass spectrometry conditions: the ion source temperature was set to 230 °C and the transfer line temperature to 280 °C. Electron ionization mode was used with an electron energy of 70 eV. The mass scan range was *m*/*z* 33 to 650, and data were acquired in full-scan mode.

The chromatographic peaks were deconvoluted using Agilent MassHunter Unknowns Analysis (version 10.1, build 10.1.733.0, Agilent Technologies). VOCs were identified by comparing them with the NIST 17 mass spectrum library and by calculating retention indices (RI) from injections of the C8–C20 n-alkane standard into the GC system under the same conditions. For filtering and validating the identified compounds, a delta RI range of −40 to 40 and a mass factor greater than 70% were used. Because no reference standards or compound-specific calibration curves were used, the detected VOCs were reported as tentative identifications, and their abundances were expressed relative to one another rather than as absolute concentrations. Therefore, differences between formulations are interpreted as variations in the relative abundance of the compounds within the analytical profiles rather than as quantitative changes in their concentrations.

### 2.4. Statistical Analysis

All statistical analyses were performed using RStudio (version 4.5.2). Volatile organic compound (VOC) data were analyzed using analysis of variance (ANOVA) to assess differences among formulations, followed by Tukey’s honestly significant difference (HSD) test for multiple comparisons. Differences were considered statistically significant at *p* < 0.05. Principal component analysis (PCA) was subsequently performed on the volatile compound data obtained by GC–MS. The results were visualized using biplots to explore relationships and differences among formulations.

## 3. Results and Discussion

### 3.1. Volatile Organic Compounds Expressed as Relative Abundances by Chemical Family

**Figure 1:**
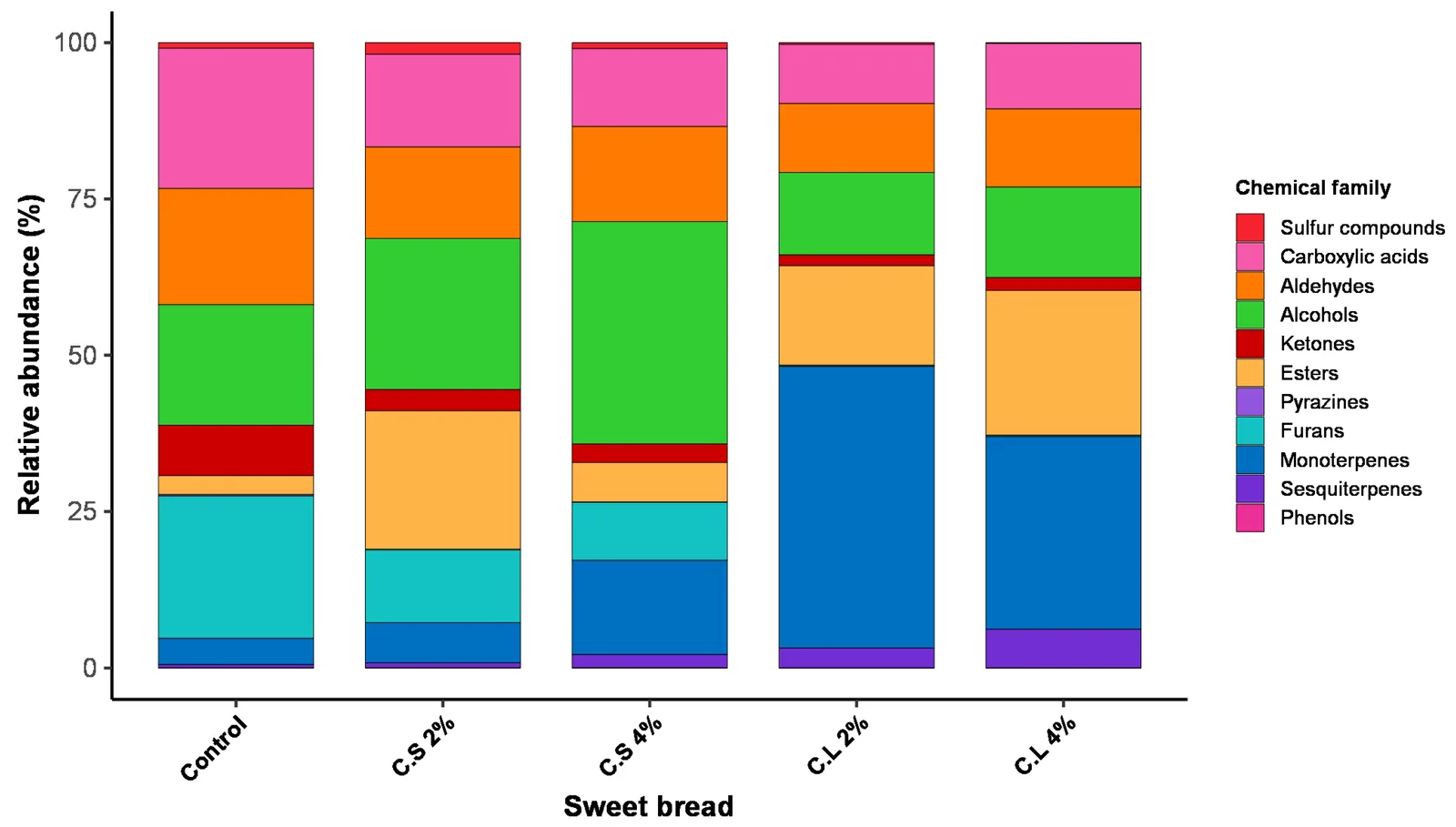
Profile of volatile compounds classified into chemical families based on the formulation of sweet breads: Note: Control, C.S. (*C. sinensis* peel flour), C.L. (*C. limetta* Risso peel flour); Volatile organic compounds expressed as relative abundances by chemical family.

The volatile compounds identified in the different sweet bread formulations totaled 165 tentatively identified constituents (Table S1, Figure 1). Figure 1 shows the relative distribution of the chemical families of the detected volatile compounds (alcohols, sulfur-containing compounds, carboxylic acids, aldehydes, ketones, esters, pyrazines, furans, monoterpenes, sesquiterpenes, and phenols). The relative abundance (%) of each compound was calculated based on its chromatographic area relative to the total area of the detected compounds. Subsequently, compounds belonging to the same chemical family were grouped by summing their relative abundances in order to determine the relative contribution of each family to each formulation. Thus, the 11 chemical families accounted for 100% of the volatile compound profile in each formulation. Each bar represents 100% of the compounds detected in each sample, while the segments represent the percentage contribution of each chemical family. In the control sample, furans (22.77%) and carboxylic acids (22.43%) accounted for the largest relative contributions, followed by aldehydes (18.61%) and pyrazines (0.21%). These compounds are generally formed during fermentation, lipid oxidation, caramelization, and Maillard reactions [8,18]. In the formulations incorporating C.S. flour, alcohols predominated (35.61%), while in the formulations with C.L. flour, monoterpenes (45.03%) and sesquiterpenes (3.18%) stood out. The greater contribution of these latter groups in the C.L. formulation could be related to the inclusion of terpenic compounds naturally present in this citrus fruit [19].

### 3.2. Analysis of Variance of Volatile Organic Compounds by Chemical Family

**Figure 2:**
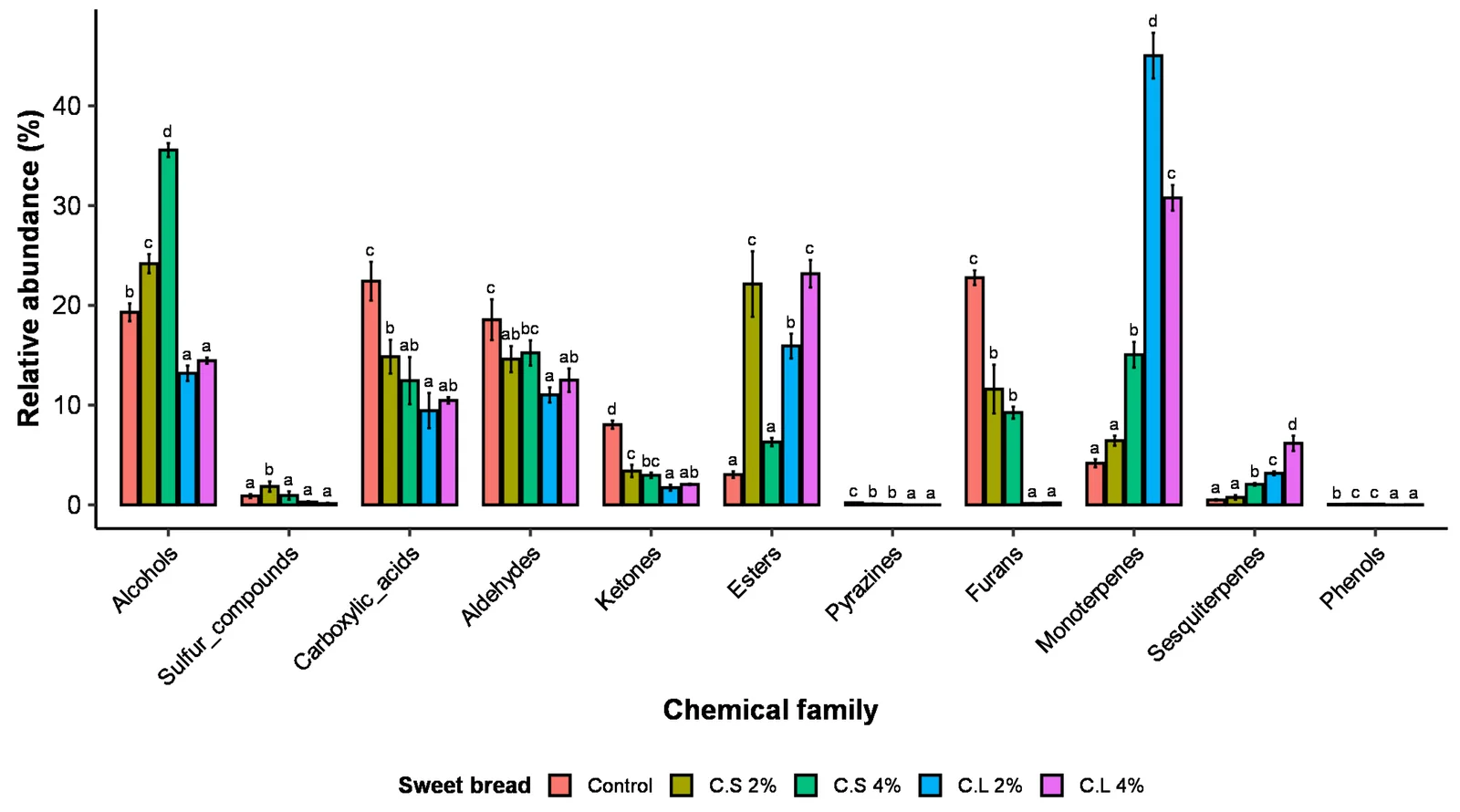
Relative abundance of chemical families of volatile compounds in sweet breads. The bars indicate the standard deviation (n = 3). Different letters indicate statistically distinct groups among the types of sweet bread according to Tukey’s test (sig. ≤ 0.05). Note: Control, C.S. (*C. sinensis* peel flour), C.L. (*C. limetta* Risso peel flour).

The profile of volatile compounds showed significant differences (*p* ≤ 0.05) among the formulations. A marked change in the chemical composition of the sweet bread was observed as a result of the incorporation of citrus flours (see Figure 2).

In the control sample, the chemical groups that showed significant differences were furans (22.772 ± 0.731%) and carboxylic acids (22.43 ± 1.944%), followed by aldehydes (18.567 ± 2.038%), ketones (8.046 ± 0.409%), and pyrazines (0.211 ± 0.017%). The addition of citrus flours caused a significant reduction in these groups, particularly furans, with a decrease in relative abundance observed in samples with 2% and 4% C.L., yielding values of 0.154 ± 0.025% and 0.208 ± 0.024%, respectively. Likewise, other groups, such as carboxylic acids, ketones, and pyrazines, tended to decrease progressively relative to the control sample. Changes in their concentrations were likely influenced by modifications to the product matrix and by the specific incorporation of the raw material, which has a characteristic volatile profile [19,20]. However, its incorporation favored the presence of terpenic compounds.

Monoterpenes increased from 4.175 ± 0.402% in the control sample to 15.055 ± 1.284% in the 4% C.S. sample, reaching a maximum of 45.029 ± 2.284% in the 2% C.L. sample, while sesquiterpenes showed lower concentrations but a more gradual increase, ranging from 0.5 ± 0.059% in the control to 6.183 ± 0.762% in C.L. 4%. This pattern suggests particular significance in formulations containing C.L., in which monoterpenes are the predominant group (Figure 1). Various studies have reported that this group is the most prevalent in citrus fruits, especially in the peels, where they account for between 91.84% and 96.7% of the total detected compounds [21,22]. However, compared with the final product, the terpenes are still present, albeit at lower concentrations. Pico et al. [11] reported that baking promotes both the release and loss of volatile compounds from the bread matrix into the oven environment. Therefore, baking conditions could significantly influence the chemical composition of the final product.

Alcohols also showed varying responses depending on the formulation. The formulations containing 2% and 4% C.S. showed a significant increase, reaching values of 24.178 ± 0.957% and 35.563 ± 0.691%, respectively, compared to the control sample (19.302 ± 0.887%), although they exhibited a lower relative abundance of aldehydes (14.612 ± 1.31% and 15.237 ± 1.259%) compared to the control. These observations could be related to a possible conversion of aldehydes into alcohols, a process in which the enzyme alcohol dehydrogenase is involved [23]. Likewise, the final composition of these chemical groups could be influenced by transformations associated with fermentation and baking [11,20].

Similarly, esters constituted another group that responded favorably to the incorporation of these flours. Their relative abundance increased from 3.039 ± 0.32% in the control to 22.136 ± 3.283% in the 2% C.S. treatment, while the 4% C.S. treatment (6.305 ± 0.413%) showed no significant differences compared to the control. Meanwhile, the formulations with 2% and 4% C.L. (15.93 ± 1.237% and 23.172 ± 1.373%, respectively) also showed a higher abundance of this group. Esters are compounds that can be formed during fermentation as a result of yeast metabolic activity or through the oxidation of lipids, primarily via the action of acetyltransferases on alcohols [11]. However, during baking, they may be lost due to heat-catalyzed hydrolysis [10,11]. Therefore, the higher abundance observed in formulations containing citrus flours could be due to a combination of their formation during fermentation and their incorporation into the formulations. However, the behavior observed in C.S. 4% would indicate that the responses of the esters were not proportional to the level of flour incorporation.

Sulfur-containing compounds are found primarily in the crust of bread, formed through non-enzymatic Maillard reactions, which involve the interaction between amino acids and sugars, leading to the formation of brown pigments (melanoidins) [8]. This group exhibited a distinctive pattern, with a significant increase in the 2% C.S. sample (1.846 ± 0.513%) compared to the control (0.9 ± 0.189%); in contrast, the phenol group remained at relatively low concentrations.

### 3.3. Chemical Families of Volatile Organic Compounds

**Figure 3:**
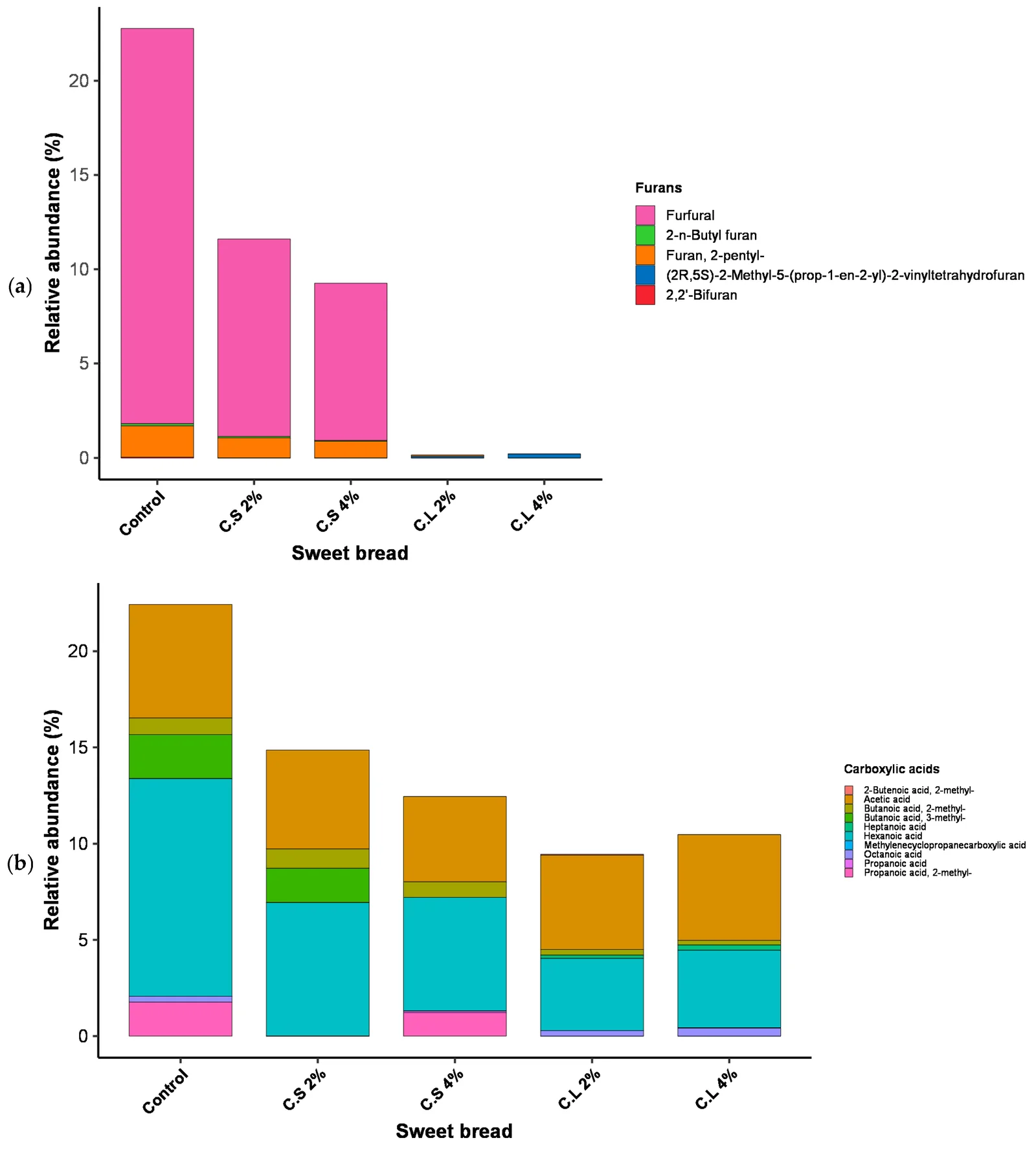

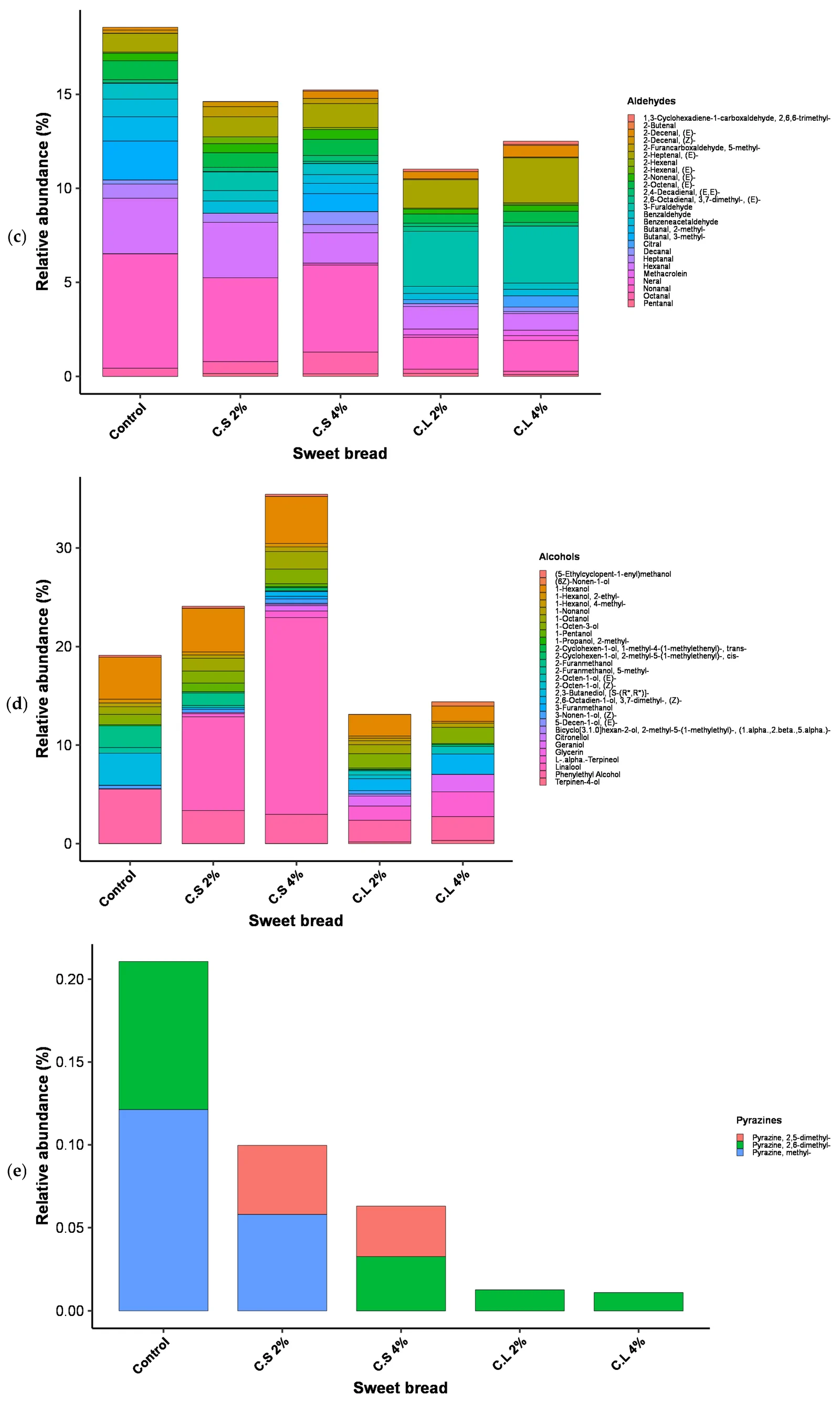

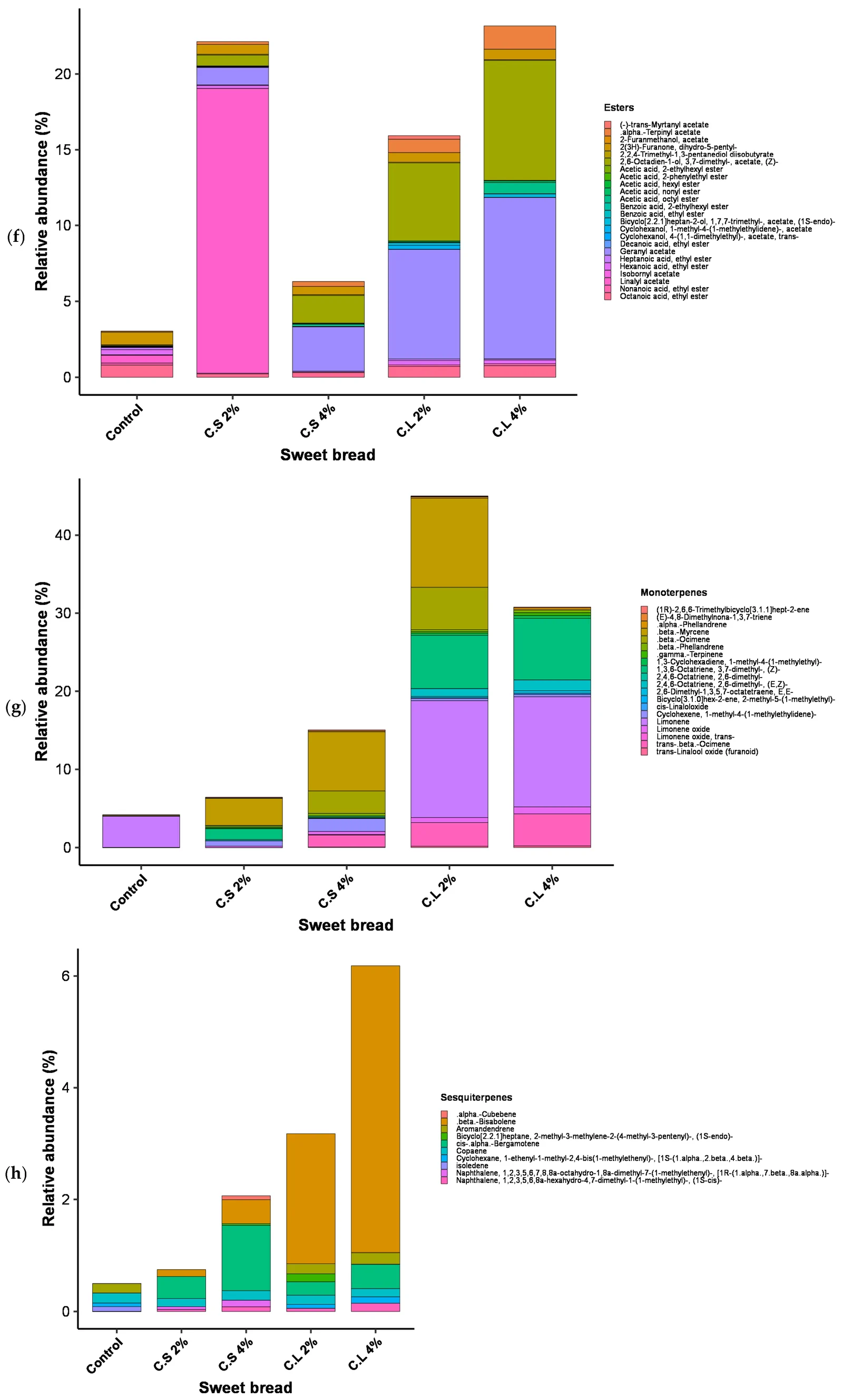
Relative abundance profile of the volatile compounds from the main chemical families present in the different sweet bread formulations: (**a**) furans; (**b**) carboxylic acids; (**c**) aldehydes; (**d**) alcohols; (**e**) pyrazines; (**f**) esters; (**g**) monoterpenes; (**h**) sesquiterpenes. Relative abundances are expressed as percentages of the total chromatographic area of the detected peaks (n = 3). Note: Control, C.S. (*C. sinensis* peel flour), C.L. (*C. limetta* Risso peel flour).

In order to further characterize the volatile profile of sweet bread formulations, the compounds corresponding to the main chemical families identified in Figure 1 were analyzed individually. Within these groups, furans, carboxylic acids, aldehydes, alcohols, esters, pyrazines, monoterpenes, and sesquiterpenes were considered because of their contributions to the volatile profile and the variations observed among formulations. Figure 3 and Table S1 show the relative abundance of the identified compounds within each of these families.

#### 3.3.1. Furans

In the furan family (Figure 3a), five compounds were identified, among which furfural and 2-pentylfuran had the highest relative abundances. Both compounds showed their highest values in the control sample, followed by C.S. 2% and C.S. 4%, while they were not detected in the formulations with C.L. 2% and C.L. 4%. The presence of these compounds may be related to mechanisms such as caramelization and Maillard reactions that occur during baking [8,24]. These observed differences could be related to changes in formulation composition and in the availability of precursors, although it is not possible to attribute this absence to a single mechanism based on the results [15].

#### 3.3.2. Carboxylic Acids

A total of 10 carboxylic acids were identified in the formulations, among which hexanoic acid, acetic acid, and 2-methylbutanoic acid stood out due to their higher relative abundances (Figure 3b). These compounds were present in all formulations, although they were most abundant in the control sample. On the other hand, heptanoic acid and octanoic acid were detected only in the C.L. 2% and C.L. 4% formulations, while propanoic acid, 2-methylpropanoic acid, and 3-methylbutanoic acid were found only in some of the C.S. 2% and C.S. 4% formulations and in the control. The presence of these compounds is consistent with findings from studies of volatile compounds in gluten-free breads and fermented doughs, in which these acids have been identified as significant components of the volatile profile [10,11,25]. These compounds are typically generated during fermentation or through non-enzymatic lipid oxidation reactions [8]. Therefore, the differences observed among formulations could be related to changes in dough composition resulting from the incorporation of different citrus flours, given that during fermentation, microbial enzymes cause biochemical changes in carbohydrates and proteins that can alter the formation of certain volatile compounds [26].

#### 3.3.3. Aldehydes

Among the 26 aldehydes identified, hexanal, heptanal, octanal, nonanal, (E)-2-heptenal, (E)-2-octenal, (E)-2-nonenal, (E)-2-decenal, (E,E)-2,4-decadienal, and benzaldehyde were present in all five sweet bread formulations, while the remaining aldehydes were present only in certain formulations (Figure 3c). The control sample contained the highest number of compounds in this family (18), the most representative of which were nonanal, hexanal, and 3-methylbutanal. The presence of these compounds is consistent with various studies reported on baked goods, which also indicate that their presence is related to different biochemical transformations occurring during bread production [10,16,26,27]. Hexanal, one of the most common compounds in the evaluated formulations, can be derived from lipid oxidation and from the fermentative metabolism of microorganisms [26], while 3-methylbutanal can form from amino acids during Strecker reactions and also as a product of the Maillard reaction during baking [18,26]. Furthermore, the formation of some aldehydes may be related to the oxidation of unsaturated acids [28]. Hexanal, (E,E)-2,4-decadienal, (E)-2-octenal, and (E)-2-heptenal are formed through the decomposition of linoleic acid, while octanal, nonanal, and (E)-2-nonenal are derived primarily from oleic acid [18,23]. Therefore, the presence of these aldehydes in all formulations would be associated with the oxidation of the lipids present in the mixture, and their absence or low production in certain formulations would be related to the ability of heterofermentative lactic acid bacteria to reduce the aldehydes to other compounds [28].

On the other hand, the addition of citrus flours positively affected the formation of new aromatic aldehydes, such as neral (0.258 ± 0.041%) and citral (0.592 ± 0.102%), in the 4% C.L. formulation (Table S1), which were not detected in the control sample. These compounds are characteristic of essential oils derived from citrus peels; therefore, their presence could be attributed to their inclusion in the formulations [29,30]. This behavior suggests that, in addition to the aldehydes generated during fermentation, these flours may have contributed their own precursors, thereby helping differentiate the aldehyde composition of the formulations.

#### 3.3.4. Alcohols

Within the alcohol family, 29 compounds were identified, of which phenylethyl alcohol, 1-hexanol, 1-octen-3-ol, 1-nonanol, and 2-ethyl-1-hexanol were the most prominent in all sweet bread formulations (Figure 3d). Phenethyl alcohol and 1-hexanol were most prevalent in the control sample, while 1-octen-3-ol was most prominent in the 4% C.L. formulation. Meanwhile, 1-nonanol and 2-ethyl-1-hexanol showed variations across the formulations, with no consistent pattern associated with the incorporation of citrus flours.

Short-chain alcohols are produced during the fermentation of sugars, while long-chain alcohols are produced through the metabolism of amino acids [18]. In this regard, studies have reported that alcohols such as 1-propanol, 2-methyl-1-propanol, 3-methyl-1-butanol, and 2-methyl-1-butanol tend to increase during the fermentation process due to the metabolic activity of yeasts [10]. Furthermore, these compounds are part of the amino acid transformation pathways described for dough fermentation, such as the formation of 3-methyl-1-butanol (isoamyl alcohol) from leucine and 2-methyl-1-propanol from valine [8], which would explain the differences observed among the sweet bread formulations.

The addition of C.S. and C.L. flours led to the formation of new aromatic alcohols, including geraniol, citronellol, linalool, terpinen-4-ol, and α-terpineol, resulting in a notable difference relative to the control sample. Their abundance depended on the type of citrus flour used. The presence of these compounds was to be expected, as they have been reported as characteristic volatile compounds of citrus peel essential oils, which vary with factors such as geographical conditions, variety, and ripeness [30,31,32]. Likewise, these compounds have been identified in breads enriched with aromatic plant materials [33], suggesting that their incorporation modifies the product’s volatile profile.

#### 3.3.5. Pyrazine

Pyrazines are volatile compounds associated with characteristic aromas in baked goods and are formed primarily during heating [34]. In this study, three pyrazines were identified: methyl pyrazine, 2,6-dimethyl pyrazine, and 2,5-dimethyl pyrazine (Figure 3e). The first two were more prevalent in the control sample, while their presence was low or undetectable in some of the C.S. and C.L. formulations. On the other hand, 2,5-dimethylpyrazine was not detected in the control sample but was detected in the formulations containing 2% and 4% C.S.

The differences observed can be attributed to the conditions under which these compounds form and are retained during baking. Ren et al. [34] note that as baking time increases, pyrazines may be present at low concentrations in the crumb, whereas they tend to be more abundant in the crust. Similarly, Nor Qhairul Izzreen et al. [35] identified 2,5-dimethylpyrazine and 2,6-dimethylpyrazine in the bread crust and reported that processing conditions can enhance the formation of Maillard reaction products. This is consistent with the identification of these types of pyrazines in sweet bread formulations.

On the other hand, the absence of pyrazines in certain formulations does not necessarily mean these compounds did not form during baking; rather, their absence may reflect their high volatility, which can lead to loss during heating [18]. Therefore, the differences observed among formulations could depend both on variations in their formation during baking and on their volatilization.

#### 3.3.6. Esters

The esters present in bread are primarily formed through esterification reactions between fatty acids and alcohols during fermentation [16]. In the present study, a total of 24 esters were detected, nine of which were present in all formulations (Figure 3f). Among them were, in particular, esters derived from carboxylic acids, such as ethyl octanoate, hexanoate, heptanoate, and nonanoate. The presence of these compounds is consistent with reports from fermented doughs and baked goods [10,16,25]. Some of these compounds may be related to yeast activity during fermentation. Pico et al. [10] reported that ethyl lactate levels increase during this stage due to yeast activity. Heating may also affect the abundance of these compounds by promoting reactions between alcohols and acids during thermal processing [36]. This explains why the presence of these components in the different sweet bread formulations is associated with the fermentation processes.

In addition to the esters mentioned above. New terpenic esters were identified as a result of the incorporation of citrus flours that were not present in the control, such as geranyl acetate; 2,6-octadien-1-ol, 3,7-dimethyl-, acetate, (Z)-; and α-terpinyl acetate, once again demonstrating their contribution to the volatile fraction of sweet bread. Some of these compounds have been reported in various studies [32,37].

#### 3.3.7. Monoterpenes and Sesquiterpenes

Monoterpene and sesquiterpene hydrocarbons generally dominate the volatile fraction of citrus fruits [21]. In the present study, these groups were particularly prominent in formulations containing C.S. and C.L. flours, consistent with the characteristic composition of citrus fruits [32]. Among the monoterpenes, 20 compounds were identified (Figure 3g). Limonene was the predominant compound in the formulations containing C.L., reaching its highest concentration of 14.957 ± 1.406% in the 2% C.L. formulation (Table S1). Furthermore, this formulation exhibited the greatest diversity of terpenoid compounds compared to the 2% C.S. and 4% C.S. formulations. Among the compounds detected, particularly in the formulations containing C.L. and C.S., were trans-β-ocimene, β-phellandrene, β-ocimene, γ-terpinene, and limonene oxide. Limonene and β-myrcene were also found in the control, although in lower abundances.

The predominance of limonene observed in formulations containing C.S. and C.L. is consistent with the literature. This compound is one of the main constituents of citrus peel essential oil and accounts for between 36 and 96 percent of the essential oil’s composition, depending on the type of citrus fruit [29]. Similarly, Toprakçı et al. [32] identified limonene and myrcene as the main volatile components of the peels of various citrus fruits, while in orange peel, they identified individual compounds such as limonene, myrcene, α-pinene, linalool, octanal, and decanal. In another study, D-limonene accounted for 67.72% in C.S. and 37.05% in C.L.; however, in the present study, the highest abundance was observed in C.L., suggesting that differences between varieties influence its abundance [19].

As for sesquiterpenes, 10 compounds were identified (Figure 3h), with copaene, aromandendrene, α-cubebene, and β-bisabolene standing out in the formulations containing C.S. and C.L. The presence of these compounds complements the terpene profile observed in the enriched formulations.

Although there are few studies that specifically evaluate the volatile compound composition in sweet breads made with C.S. and C.L. flours, the presence of monoterpenes and sesquiterpenes in the final product may be related to their contribution from the raw material. In this regard, the detection of these compounds after baking suggests that a fraction of them was retained following thermal processing. This behavior is of great interest because citrus fruits are an important source of bioactive compounds; furthermore, studies on baked goods have indicated that incorporating citrus peel flours can modify the phenolic composition and antioxidant activity of the product, which are closely related to its volatile composition [6,19,38,39].

### 3.4. Multivariate Analysis

**Figure 4:**
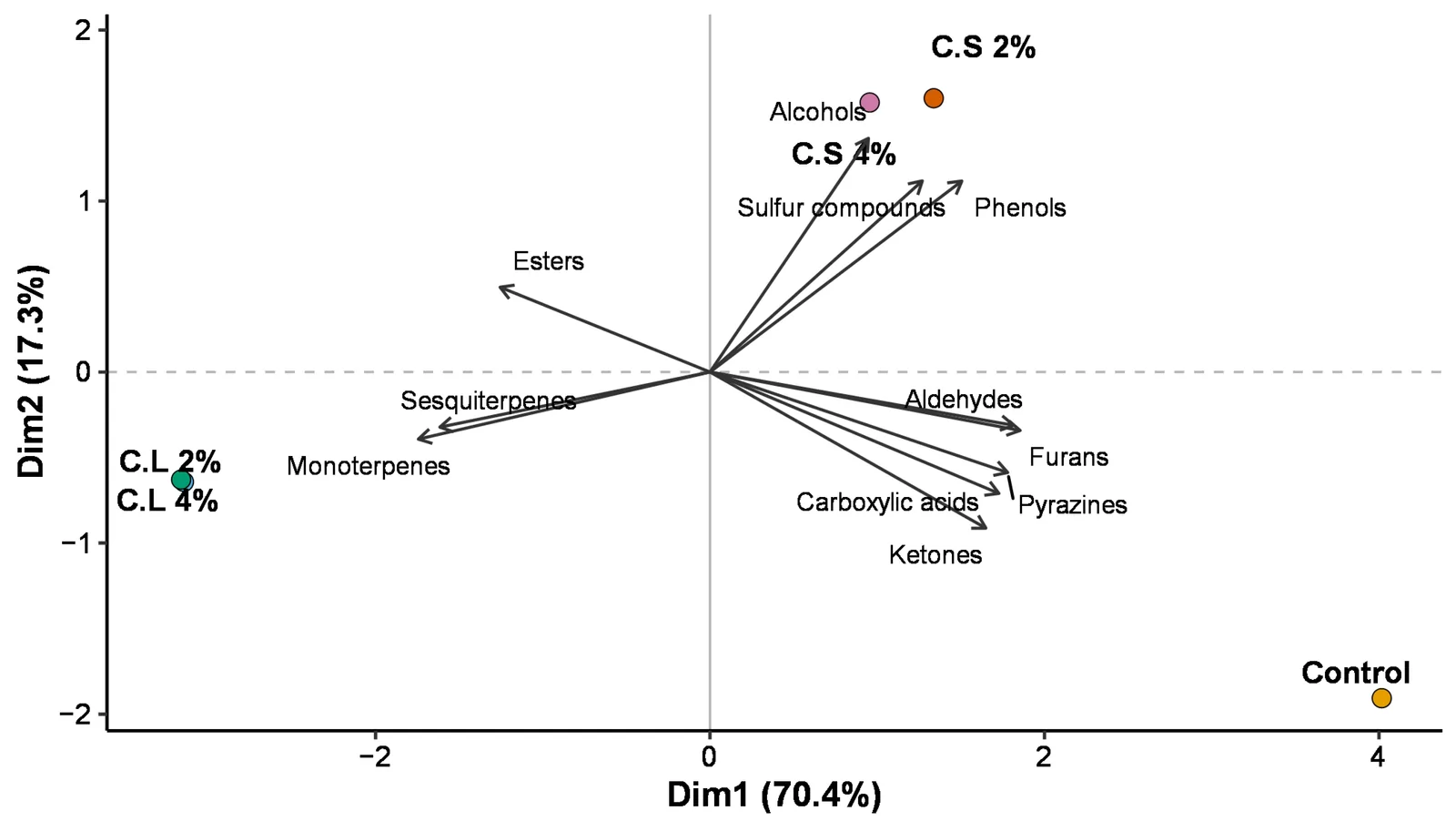
Principal component biplot of sweet bread formulations and the chemical families of volatile compounds. Note: Control, C.S. (*C. sinensis* peel flour), C.L. (*C. limetta* Risso peel flour).

To visualize the differences between the formulations based on their volatile compound profiles, a principal component analysis (PCA) was performed (Figure 4). The first principal component explained 70.4% of the variance, and the second principal component explained 17.3%, accounting for 87.7% of the total variability. It is clearly observed that the formulations with C.L. 2% and C.L. 4% are close to each other, indicating a similarity in their volatile profile (monoterpenes and sesquiterpenes), whereas C.S. 2% and C.S. 4% were located in the positive region of Dim1 and exhibited a different chemical profile, with greater proximity to the families of alcohols, phenols, and sulfur-containing compounds. In contrast, the control sample was distinct from the formulations containing C.L. and C.S. flours, showing an association with aldehydes, furans, pyrazines, carboxylic acids, and ketones. In this regard, the PCA distribution is consistent with previously identified differences in chemical family composition, suggesting that incorporating these flours modified the volatile profile of the sweet breads.

## 4. Conclusions

The sweet bread formulations made with flours from the peels of *Citrus limetta* Risso and *Citrus sinensis* showed differences in their volatile composition, evidenced primarily by the distribution of the identified chemical families. *Citrus limetta* Risso peel flour showed a greater contribution of monoterpenes and sesquiterpenes, with the 2% formulation presenting the highest relative abundance of monoterpenes, whereas the 4% formulation showed a progressive increase in sesquiterpenes. In contrast, increasing the proportion of citrus peel flour was associated with changes in several other volatile families, indicating that the two citrus sources exerted differentiated effects on the volatile profile. Among the formulations evaluated, the 2% *Citrus limetta* Risso treatment exhibited the most distinctive terpene-rich profile, suggesting that this inclusion level may be particularly suitable for exhibiting a distinctive terpene-rich volatile profile.

Principal component analysis explained 87.9% of the total variability and enabled differentiation of the formulations based on their volatile compound composition. These results show that citrus species and incorporation level are relevant factors in characterizing the chemical differences among the sweet bread formulations studied. However, the results are limited to the evaluated incorporation levels and do not permit the establishment of a universal optimal level.

Future research should evaluate the stability of characteristic volatile compounds during storage and their dynamic changes throughout shelf life, optimize key technological parameters, and assess the industrial adaptability and process performance of citrus peel flour-based bread formulations.

## Figures and Tables

**Table 1:** Ingredients Used in the Preparation of Sweet Breads.

Ingredients in 100 g	Control	C.S. 2%	C.S. 4%	C.L. 2%	C.L. 4%
Wheat flour (g)	49.83	48.84	47.83	48.84	47.83
Citrus peel flour (g)	0.00	1.00	2.00	1.00	2.00
Sugar (g)	16.94	16.94	16.94	16.94	16.94
Vegetable shortening (g)	4.98	4.98	4.98	4.98	4.98
Freeze-dried dry yeast (g)	1.66	1.66	1.66	1.66	1.66
Eggs (g)	6.64	6.64	6.64	6.64	6.64
Water (mL)	19.93	19.93	19.93	19.93	19.93

## Data Availability

The original contributions presented in this study are included in the article. Further inquiries can be directed to the corresponding author.

## Supplementary Materials

The following supporting information can be downloaded at https://www.mdpi.com/article/10.3390/foods15213931/s1. Table S1: Relative abundance (%) of the volatile organic compounds identified in sweet breads.
