# Supplementary material for: Volatile Profiles of Sweet Breads Enriched with Orange and Lime Peel Flours
Source: Foods. 2026 Nov 2;15(21):3931. doi: 10.3390/foods15213931 (PMC13763299; doi:10.3390/foods15213931)
Supplement: Supplementary file 1 [file foods-15-03931-s001.zip › foods-4584164-supplementary.pdf]

Table S1. Relative abundance (%) of the volatile organic compounds identified in sweet breads.

RT (min)	Compound name	RI	LRI	Delta RI	Match Factor	Control	C.S 2%	C.S 4%	C. L 2%	C. L 4%
	**Sulfur compounds**									
7.02	Carbon disulfide	579	549	-28.250	85.588	0.421 ± 0.177 [a]	1.246 ± 0.328 [b]	0.596 ± 0.357 [a]	0.118 ± 0.079 [a]	ND
15.46	Disulfide, dimethyl	748	746	-2.178	94.367	ND	0.16 ± 0.078 [b]	0.089 ± 0.038 [ab]	0.05 ± 0.009 [a]	0.046 ± 0.023 [a]
23.35	Dimethyl sulfone	907	922	15.738	78.221	0.022 ± 0.011 [b]	ND	0.01 ± 0.003 [ab]	ND	ND
23.41	Methional	908	907	-0.623	91.588	0.418 ± 0.015 [d]	0.231 ± 0.05 [c]	0.131 ± 0.049 [b]	0.056 ± 0.035 [ab]	0.015 ± 0.01 [a]
27.14	Dimethyl trisulfide	987	970	-16.722	93.290	0.039 ± 0.007 [a]	0.208 ± 0.168 [a]	0.124 ± 0.073 [a]	0.066 ± 0.011 [a]	0.069 ± 0.042 [a]
	**Carboxylic acids**									
7.85	Acetic acid	595	610	15.268	98.878	5.902 ± 1.094 [a]	5.129 ± 0.712 [a]	4.438 ± 0.959 [a]	4.91 ± 0.845 [a]	5.51 ± 0.233 [a]
11.44	Propanoic acid	667	700	30.659	76.746	ND	ND	0.095 ± 0.086 [a]	ND	ND
14.80	Propanoic acid, 2-methyl-	735	774	38.540	86.858	1.767 ± 0.31 [c]	ND	1.215 ± 0.11 [b]	ND	ND
19.21	Butanoic acid, 3-methyl-	823	863	39.875	94.462	2.267 ± 0.141 [c]	1.791 ± 0.154 [b]	ND	ND	ND
19.67	Butanoic acid, 2-methyl-	832	861	28.115	82.836	0.869 ± 0.106 [b]	1.001 ± 0.168 [b]	0.804 ± 0.16 [b]	0.296 ± 0.1 [a]	0.234 ± 0.004 [a]
20.48	Methylenecyclopropanecarboxylic acid	849	844	-5.030	86.122	ND	ND	ND	ND	0.029 ± 0.011 [b]
22.83	2-Butenoic acid, 2-methyl-	896	928	13.191	84.355	ND	ND	ND	0.04 ± 0.022 [b]	ND
25.97	Hexanoic acid	962	990	26.565	93.072	11.319 ± 0.518 [c]	6.938 ± 1.193 [b]	5.902 ± 1.158 [ab]	3.759 ± 0.788 [a]	4.021 ± 0.083 [a]
30.15	Heptanoic acid	1055	1078	22.983	84.963	ND	ND	ND	0.169 ± 0.016 [b]	0.273 ± 0.047 [c]
34.25	Octanoic acid	1153	1180	28.113	95.958	0.306 ± 0.047 [b]	ND	ND	0.28 ± 0.057 [b]	0.412 ± 0.04 [c]
	**Aldehydes**									
12.82	Pentanal	695	699	3.898	92.993	ND	0.153 ± 0.033 [b]	0.135 ± 0.021 [b]	0.163 ± 0.067 [b]	0.097 ± 0.013 [b]
17.98	Hexanal	799	800	1.799	89.432	2.961 ± 0.268 [a]	2.95 ± 1.285 [a]	1.621 ± 1.345 [a]	1.19 ± 0.297 [a]	0.881 ± 0.138 [a]
23.09	Heptanal	901	901	0.280	93.854	0.759 ± 0.113 [c]	0.487 ± 0.163 [b]	0.439 ± 0.045 [b]	0.164 ± 0.028 [a]	0.108 ± 0.015 [a]
27.89	Octanal	1003	1003	-0.231	97.622	0.434 ± 0.022 [b]	0.637 ± 0.033 [c]	1.155 ± 0.089 [d]	0.224 ± 0.037 [a]	0.181 ± 0.022 [a]
32.35	Nonanal	1106	1104	-1.601	98.843	6.077 ± 0.943 [c]	4.449 ± 0.763 [b]	4.634 ± 0.286 [bc]	1.687 ± 0.138 [a]	1.631 ± 0.069 [a]
36.47	Decanal	1208	1206	-1.802	88.515	0.215 ± 0.061 [b]	ND	0.683 ± 0.014 [c]	ND	0.243 ± 0.013 [b]
7.66	Methacrolein	592	567	-24.564	83.194	ND	ND	ND	0.31 ± 0.076 [b]	0.286 ± 0.053 [b]
10.73	2-Butenal	653	629	-23.081	84.511	ND	ND	ND	ND	0.057 ± 0.048 [b]
20.70	2-Hexenal	853	851	-2.575	94.546	ND	ND	0.11 ± 0.015 [c]	ND	0.066 ± 0.004 [b]
20.72	2-Hexenal, (E)-	853	854	0.611	88.898	0.068 ± 0.003 [a]	0.364 ± 0.094 [b]	ND	0.05 ± 0.006 [a]	0.074 ± 0.012 [a]

RT (min)	Compound name	RI	LRI	Delta RI	Match Factor	Control	C.S 2%	C.S 4%	C. L 2%	C. L 4%
25.79	2-Heptenal, (E)-	958	958	-0.548	93.870	0.992 ± 0.044 [a]	1.07 ± 0.181 [a]	1.268 ± 0.105 [a]	1.512 ± 0.246 [a]	2.392 ± 0.471 [b]
30.41	2-Octenal, (E)-	1061	1060	-1.082	85.296	1.011 ± 0.237 [b]	0.78 ± 0.266 [ab]	0.863 ± 0.108 [ab]	0.491 ± 0.03 [a]	0.595 ± 0.039 [ab]
34.71	2-Nonenal, (E)-	1164	1162	-1.919	97.456	0.395 ± 0.038 [abc]	0.477 ± 0.144 [bc]	0.526 ± 0.029 [c]	0.263 ± 0.015 [a]	0.312 ± 0.006 [ab]
38.73	2-Decenal, (E)-	1267	1263	-4.104	94.003	0.151 ± 0.013 [b]	ND	0.395 ± 0.016 [c]	0.388 ± 0.026 [c]	0.617 ± 0.034 [d]
38.71	2-Decenal, (Z)-	1267	1252	-15.043	77.250	0.164 ± 0.012 [b]	0.266 ± 0.106 [b]	ND	ND	ND
40.90	2,4-Decadienal, (E,E)-	1326	1317	-9.498	91.685	0.163 ± 0.011 [a]	0.223 ± 0.05 [ab]	0.307 ± 0.039 [b]	0.176 ± 0.027 [a]	0.192 ± 0.035 [a]
10.87	Butanal, 3-methyl-	656	652	-5.291	82.727	2.071 ± 0.525 [c]	ND	0.962 ± 0.343 [b]	ND	ND
11.29	Butanal, 2-methyl-	664	662	-4.004	90.326	1.291 ± 0.497 [b]	ND	0.547 ± 0.276 [a]	ND	ND
18.68	3-Furaldehyde	812	832	19.259	86.169	0.033 ± 0.01 [a]	0.986 ± 0.683 [a]	0.015 ± 0.002 [a]	2.928 ± 0.409 [b]	3.033 ± 0.287 [b]
26.05	2-Furancarboxaldehyde, 5-methyl-	964	965	39.017	82.647	0.007 ± 0.003 [a]	0.538 ± 0.109 [c]	0.271 ± 0.027 [b]	0.045 ± 0.016 [a]	0.04 ± 0.007 [a]
36.87	1,3-Cyclohexadiene-1-carboxaldehyde, 2,6,6-trimethyl-	1219	1201	-17.714	94.979	ND	ND	0.062 ± 0.009 [b]	0.133 ± 0.019 [c]	0.169 ± 0.014 [d]
38.95	2,6-Octadienal, 3,7-dimethyl-, (E)-	1273	1270	-3.543	93.336	ND	0.024 ± 0.005 [a]	0.108 ± 0.005 [b]	0.253 ± 0.069 [c]	ND
26.46	Benzaldehyde	973	962	-10.577	98.696	0.832 ± 0.096 [d]	0.556 ± 0.115 [bc]	0.585 ± 0.054 [c]	0.381 ± 0.049 [ab]	0.336 ± 0.018 [a]
30.11	Benzeneacetaldehyde	1054	1045	-9.143	85.564	0.941 ± 0.059 [b]	0.65 ± 0.119 [ab]	0.459 ± 0.083 [a]	0.324 ± 0.17 [a]	0.347 ± 0.212 [a]
37.93	Neral	1246	1240	-6.425	90.564	ND	ND	0.093 ± 0.008 [b]	0.13 ± 0.014 [b]	0.258 ± 0.041 [c]
38.96	Citral	1274	1276	2.254	92.571	ND	ND	ND	0.213 ± 0.15 [a]	0.592 ± 0.102 [b]
	**Alcohols**									
9.66	1-Propanol, 2-methyl-	632	625	-5.450	94.607	0.121 ± 0.009 [b]	ND	0.122 ± 0.014 [b]	ND	ND
16.17	1-Pentanol	762	765	3.030	98.347	ND	0.894 ± 0.26 [b]	0.283 ± 0.026 [a]	0.135 ± 0.027 [a]	ND
16.74	2,3-Butanediol, [S-(R*,R*)]-	774	743	-31.130	85.823	3.268 ± 0.434 [b]	ND	ND	ND	ND
20.48	2-Furanmethanol	849	859	9.991	98.343	2.202 ± 0.589 [c]	1.264 ± 0.357 [b]	ND	0.089 ± 0.059 [a]	ND
20.51	3-Furanmethanol	849	885	35.688	97.042	ND	ND	0.27 ± 0.051 [b]	ND	ND
21.30	1-Hexanol	865	868	2.954	99.357	4.273 ± 0.369 [b]	4.415 ± 0.558 [b]	4.765 ± 0.368 [b]	2.205 ± 0.097 [a]	1.561 ± 0.151 [a]
25.31	2-Furanmethanol, 5-methyl-	948	958	9.746	96.267	0.568 ± 0.074 [c]	0.209 ± 0.065 [b]	0.078 ± 0.055 [ab]	ND	ND
25.69	Glycerin	956	967	10.679	84.145	ND	0.104 ± 0.015 [b]	ND	ND	ND
26.20	1-Hexanol, 4-methyl-	967	953	-14.143	73.327	ND	ND	ND	0.275 ± 0.101 [b]	ND
26.73	1-Octen-3-ol	978	980	1.683	96.842	1.054 ± 0.059 [a]	1.224 ± 0.107 [a]	1.493 ± 0.068 [a]	1.457 ± 0.312 [a]	1.666 ± 0.447 [a]
28.92	1-Hexanol, 2-ethyl-	1027	1030	3.018	94.920	0.388 ± 0.201 [a]	0.31 ± 0.126 [a]	0.358 ± 0.173 [a]	0.205 ± 0.08 [a]	0.179 ± 0.009 [a]
30.64	2-Octen-1-ol, (E)-	1066	1067	0.597	89.374	ND	ND	ND	0.424 ± 0.147 [b]	0.76 ± 0.185 c

RT (min)	Compound name	RI	LRI	Delta RI	Match Factor	Control	C.S 2%	C.S 4%	C. L 2%	C. L 4%
30.65	2-Octen-1-ol, (Z)-	1066	1067	0.596	87.955	ND	ND	ND	0.375 ± 0.01 [b]	ND
30.72	1-Octanol	1068	1071	2.901	88.499	0.769 ± 0.033 [b]	1.296 ± 0.475 [bc]	1.786 ± 0.134 [c]	0.918 ± 0.15 [b]	ND
31.35	(5-Ethylcyclopent-1-enyl)methanol	1083	1073	-9.532	87.996	0.198 ± 0.014 [b]	0.205 ± 0.022 [b]	0.226 ± 0.011 [b]	ND	ND
31.36	Bicyclo[3.1.0]hexan-2-ol, 2-methyl-5-(1-methylethyl)-, (1.alpha.,2.beta.,5.alpha.)-	1082	1070	-12.639	76.357	ND	ND	ND	0.204 ± 0.006 [b]	ND
32.17	Linalool	1101	1099	-8.287	98.590	ND	9.53 ± 1.314 [b]	19.971 ± 1.389 [c]	ND	ND
33.10	Phenylethyl Alcohol	1124	1116	-8.403	98.599	5.549 ± 0.175 [c]	3.346 ± 0.46 [b]	2.962 ± 0.44 [ab]	2.193 ± 0.28 [a]	2.438 ± 0.136 [a]
33.57	2-Cyclohexen-1-ol, 1-methyl-4-(1-methylethenyl)-, trans-	1136	1123	-12.901	82.179	ND	ND	ND	0.063 ± 0.011 [b]	0.101 ± 0.026 [c]
34.40	(6Z)-Nonen-1-ol	1156	1171	14.530	88.779	ND	ND	ND	ND	0.431 ± 0.034 [b]
34.40	3-Nonen-1-ol, (Z)-	1156	1156	-0.274	90.690	0.363 ± 0.06 [b]	0.343 ± 0.086 [b]	0.439 ± 0.035 [b]	0.348 ± 0.055 [b]	ND
34.94	1-Nonanol	1170	1173	3.301	95.530	0.376 ± 0.051 [a]	0.325 ± 0.116 [a]	0.459 ± 0.03 [a]	0.388 ± 0.056 [a]	0.411 ± 0.06 [a]
36.12	Terpinen-4-ol	1199	1177	-21.999	81.737	ND	ND	ND	0.174 ± 0.011 [b]	0.307 ± 0.115 [b]
36.59	L-.alpha.-Terpineol	1211	1190	-20.943	97.916	ND	0.315 ± 0.056 [b]	0.663 ± 0.018 [c]	1.446 ± 0.034 [d]	2.504 ± 0.117 [e]
37.18	Citronellol	1227	1228	1.236	85.790	ND	ND	0.149 ± 0.02 [b]	ND	ND
37.32	2,6-Octadien-1-ol, 3,7-dimethyl-, (Z)-	1230	1228	-2.392	96.026	ND	0.174 ± 0.044 [ab]	0.483 ± 0.026 [b]	1.213 ± 0.068 c	2.073 ± 0.276 [d]
37.42	2-Cyclohexen-1-ol, 2-methyl-5-(1-methylethenyl)-, cis-	1233	1229	-3.946	92.980	ND	0.123 ± 0.013 [b]	0.314 ± 0.032 [d]	ND	0.192 ± 0.008 [c]
38.23	Geraniol	1254	1255	1.034	98.797	ND	ND	0.547 ± 0.071 [b]	1.004 ± 0.045 [c]	1.767 ± 0.232 [d]
38.42	5-Decen-1-ol, (E)-	1259	1266	5.556	75.617	ND	ND	0.088 ± 0.019 [b]	ND	ND
	**Ketones**									
8.19	2,3-Butanedione	602	595	-7.942	88.821	ND	ND	0.181 ± 0.029 [c]	0.14 ± 0.018 [bc]	0.13 ± 0.025 [b]
11.09	2-Propanone, 1-hydroxy-	660	665	4.595	77.938	ND	0.215 ± 0.02 [b]	ND	ND	ND
12.66	2,3-Pentanedione	692	698	7.098	94.877	0.409 ± 0.114 [b]	0.09 ± 0.027 [a]	0.12 ± 0.032 [a]	0.024 ± 0.002 [a]	ND
13.26	Acetoin	704	713	9.229	96.015	5.369 ± 0.539 [b]	0.869 ± 0.052 [a]	0.679 ± 0.152 [a]	0.955 ± 0.192 [a]	0.952 ± 0.026 [a]
20.93	2-Propanone, 1-(acetyloxy)-	858	870	12.796	89.679	0.214 ± 0.016 [d]	0.07 ± 0.015 [c]	0.032 ± 0.003 [b]	ND	ND
22.17	4-Cyclopentene-1,3-dione	882	881	-1.266	93.869	0.109 ± 0.024 [c]	0.063 ± 0.021 [b]	0.026 ± 0.007 [ab]	ND	ND
22.25	3-Heptanone	884	887	2.872	78.883	ND	ND	0.007 ± 0.004 [b]	ND	ND
22.43	2-Heptanone	888	891	3.436	97.793	0.151 ± 0.051 [b]	0.133 ± 0.065 [b]	0.127 ± 0.011 [b]	ND	ND

RT (min)	Compound name	RI	LRI	Delta RI	Match Factor	Control	C.S 2%	C.S 4%	C. L 2%	C. L 4%
23.57	Ethanone, 1-(2-furanyl)-	911	911	-0.158	97.780	1.229 ± 0.145 ^{c}	0.562 ± 0.127 ^{b}	0.404 ± 0.011 ^{b}	0.111 ± 0.023 ^{a}	0.105 ± 0.015 ^{a}
25.54	2(3H)-Furanone, dihydro-5-methyl-	953	958	4.765	82.623	0.036 ± 0.013 ^{b}	ND	ND	ND	ND
26.81	2,3-Octanedione	980	984	3.812	87.974	ND	ND	ND	ND	0.192 ± 0.018 ^{b}
26.97	5-Hepten-2-one, 6-methyl-	983	986	2.517	93.762	ND	0.235 ± 0.056 ^{b}	0.205 ± 0.042 ^{b}	0.165 ± 0.023 ^{b}	0.17 ± 0.05 ^{b}
27.22	2-Octanone	989	990	1.263	73.662	ND	ND	0.103 ± 0.017 ^{b}	ND	ND
28.21	1-Propanone, 1-(2-furanyl)-	1011	1011	0.243	77.479	0.035 ± 0.012 ^{b}	ND	ND	ND	ND
29.13	3(2H)-Furanone, 4-hydroxy-5-methyl-	1032	1054	21.913	75.235	ND	0.009 ± 0.003 ^{b}	ND	ND	ND
29.43	3-Octen-2-one	1039	1040	0.909	73.153	ND	ND	0.06 ± 0.013 ^{b}	ND	ND
30.27	2(3H)-Furanone, 5-ethyldihydro-	1058	1057	-0.885	90.765	ND	0.216 ± 0.018 ^{b}	0.235 ± 0.095 ^{b}	0.088 ± 0.017 ^{a}	0.081 ± 0.008 ^{a}
30.47	3,6-Heptanedione	1062	1041	-21.410	82.066	ND	0.207 ± 0.033 ^{b}	ND	ND	ND
30.55	Ethanone, 1-(1H-pyrrol-2-yl)-	1064	1064	-0.246	80.166	0.077 ± 0.003 ^{c}	ND	ND	0.018 ± 0.006 ^{b}	ND
31.16	Acetophenone	1078	1065	-13.180	90.421	0.058 ± 0.009 ^{a}	0.027 ± 0.026 ^{a}	0.043 ± 0.021 ^{a}	0.028 ± 0.016 ^{a}	0.044 ± 0.005 ^{a}
31.68	2-Nonanone	1090	1092	1.874	73.908	ND	ND	0.059 ± 0.024 ^{b}	ND	ND
31.69	2-Nonanone, 3-(hydroxymethyl)-	1090	1093	2.567	75.230	ND	ND	ND	0.019 ± 0.003 ^{b}	ND
34.30	4H-Pyran-4-one, 2,3-dihydro-3,5-dihydroxy-6-methyl-	1154	1151	-3.046	88.900	0.144 ± 0.008 ^{a}	0.381 ± 0.32 ^{a}	0.089 ± 0.013 ^{a}	ND	ND
35.04	Camphor	1172	1145	-27.210	77.006	0.058 ± 0.038 ^{b}	ND	ND	ND	ND
36.74	Cyclohexanone, 2-methyl-5-(1-methylethenyl)-, trans-	1215	1201	-21.207	85.568	ND	ND	0.082 ± 0.009 ^{b}	ND	ND
37.85	4-Methyleneisophorone	1244	1242	-2.552	87.689	ND	0.06 ± 0.008 ^{b}	0.119 ± 0.011 ^{c}	ND	ND
38.52	D-Carvone	1262	1246	-15.968	87.457	ND	ND	0.082 ± 0.004 ^{b}	ND	0.145 ± 0.008 ^{c}
38.52	Carvone	1262	1242	-20.466	86.509	ND	0.036 ± 0.006 ^{b}	0.087 ± 0.012 ^{c}	0.086 ± 0.014 ^{c}	0.135 ± 0.006 ^{d}
38.87	Cyclohexanone, 2-butyl-	1271	1250	-21.359	85.983	0.156 ± 0.016 ^{b}	0.174 ± 0.012 ^{bc}	0.191 ± 0.01 ^{c}	0.089 ± 0.013 ^{a}	0.099 ± 0.012 ^{a}
39.55	2-Cyclohexen-1-one, 3-methyl-6-(1-methylethenyl)-	1289	1272	-17.502	83.880	ND	0.008 ± 0.002 ^{b}	0.022 ± 0.001 ^{c}	ND	ND
40.84	Ethanone, 1-(2-hydroxy-5-methylphenyl)-	1325	1316	-9.075	75.280	ND	0.04 ± 0.019 ^{b}	0.021 ± 0.004 ^{ab}	ND	ND
	**Esters**									
27.17	2-Furanmethanol, acetate	988	995	7.172	73.892	0.077 ± 0.03 ^{b}	ND	ND	ND	ND
27.44	Hexanoic acid, ethyl ester	993	1000	6.344	90.022	0.336 ± 0.084 ^{b}	0.19 ± 0.076 ^{ab}	ND	0.284 ± 0.146 ^{b}	0.246 ± 0.012 ^{b}

RT (min)	Compound name	RI	LRI	Delta RI	Match Factor	Control	C.S 2%	C.S 4%	C. L 2%	C. L 4%
28.03	Acetic acid, hexyl ester	1007	1011	4.302	78.636	0.033 ± 0.008 a	0.031 ± 0.007 a	0.048 ± 0.012 a	0.062 ± 0.026 a	0.032 ± 0.003 a
31.78	Heptanoic acid, ethyl ester	1092	1097	4.627	91.318	0.142 ± 0.036 c	0.044 ± 0.01 a	0.054 ± 0.005 ab	0.103 ± 0.027 bc	0.081 ± 0.01 ab
33.86	Acetic acid, 2-ethylhexyl ester	1143	1129	-14.310	81.566	ND	ND	0.024 ± 0.002 b	ND	ND
35.40	Benzoic acid, ethyl ester	1181	1171	-10.201	87.297	0.014 ± 0.002 ab	0.015 ± 0.003 ab	0.017 ± 0.003 b	0.009 ± 0.003 a	0.012 ± 0.003 ab
35.82	Octanoic acid, ethyl ester	1191	1196	4.557	98.402	0.813 ± 0.147 b	0.229 ± 0.05 a	0.307 ± 0.031 a	0.713 ± 0.163 b	0.77 ± 0.078 b
36.35	Acetic acid, octyl ester	1205	1210	5.261	89.763	ND	ND	0.148 ± 0.022 b	ND	0.741 ± 0.048 c
38.05	Linalyl acetate	1249	1257	7.523	96.919	0.516 ± 0.083 a	18.777 ± 2.904 b	ND	ND	ND
38.54	Acetic acid, 2-phenylethyl ester	1263	1258	-4.827	89.628	0.028 ± 0.006 b	ND	ND	ND	ND
39.61	Nonanoic acid, ethyl ester	1291	1296	5.243	94.424	0.121 ± 0.018 b	0.044 ± 0.009 a	0.046 ± 0.002 a	0.105 ± 0.033 b	0.124 ± 0.03 b
40.04	Bicyclo[2.2.1]heptan-2-ol, 1,7,7-trimethyl-, acetate, (1S-endo)-	1302	1284	-18.397	95.031	ND	0.019 ± 0.005 a	ND	0.163 ± 0.017 b	0.234 ± 0.011 c
40.10	Acetic acid, nonyl ester	1304	1308	3.595	86.676	ND	ND	ND	0.045 ± 0.002 b	0.104 ± 0.03 c
40.19	Cyclohexanol, 4-(1,1-dimethylethyl)-, acetate, trans-	1307	1322	15.416	77.674	0.029 ± 0.01 b	0.03 ± 0.007 b	ND	ND	ND
40.23	Isobornyl acetate	1308	1286	-21.553	72.875	0.033 ± 0.023 b	ND	ND	ND	ND
41.54	Cyclohexanol, 1-methyl-4-(1-methylethylidene)-, acetate	1345	1341	-3.536	77.083	ND	ND	ND	0.265 ± 0.022 b	ND
41.63	(-)-trans-Myrtanyl acetate	1347	1387	39.818	88.386	ND	ND	ND	0.235 ± 0.022 b	ND
41.97	2,6-Octadien-1-ol, 3,7-dimethyl-, acetate, (Z)-	1357	1364	7.300	98.889	ND	0.709 ± 0.097 a	1.812 ± 0.159 b	5.132 ± 0.384 c	7.943 ± 0.522 d
42.09	.alpha.-Terpinyl acetate	1360	1350	-10.179	96.812	ND	0.185 ± 0.078 b	0.329 ± 0.015 b	0.883 ± 0.029 c	1.539 ± 0.117 d
42.53	2(3H)-Furanone, dihydro-5-pentyl-	1373	1363	-9.595	96.404	0.82 ± 0.066 b	0.647 ± 0.171 ab	0.534 ± 0.053 a	0.657 ± 0.078 ab	0.684 ± 0.04 ab
42.63	Geranyl acetate	1375	1382	6.416	99.372	ND	1.146 ± 0.135 b	2.918 ± 0.264 c	7.215 ± 0.492 d	10.612 ± 0.53 e
43.16	Decanoic acid, ethyl ester	1390	1396	5.648	76.422	0.022 ± 0.006 b	ND	ND	ND	ND
49.90	2,2,4-Trimethyl-1,3-pentanediol diisobutyrate	1597	1588	-9.769	86.798	0.049 ± 0.017 a	0.054 ± 0.019 a	0.059 ± 0.002 a	0.05 ± 0.006 a	0.043 ± 0.004 a
53.68	Benzoic acid, 2-ethylhexyl ester	1725	1735	10.442	70.738	0.007 ± 0.004 a	0.015 ± 0.009 a	0.01 ± 0.002 a	0.011 ± 0.001 a	0.007 ± 0.001 a
	**Pyrazines**									
19.34	Pyrazine, methyl-	826	831	4.811	81.867	0.121 ± 0.015 c	0.058 ± 0.027 b	ND	ND	ND
23.74	Pyrazine, 2,6-dimethyl-	915	917	1.931	93.488	0.089 ± 0.002 d	ND	0.033 ± 0.003 c	0.013 ± 0.003 b	0.011 ± 0.001 b

RT (min)	Compound name	RI	LRI	Delta RI	Match Factor	Control	C.S 2%	C.S 4%	C. L 2%	C. L 4%
23.77	Pyrazine, 2,5-dimethyl-	915	917	1.514	84.379	ND	0.042 ± 0.007 [b]	0.03 ± 0.006 [b]	ND	ND
	**Furans**									
19.68	Furfural	833	833	0.356	97.919	20.962 ± 0.722 [c]	10.471 ± 2.3 [b]	8.331 ± 0.49 [b]	ND	ND
22.66	2-n-Butyl furan	892	893	0.521	87.341	0.113 ± 0.051 [c]	0.076 ± 0.022 [bc]	0.038 ± 0.011 [ab]	ND	ND
27.40	Furan, 2-pentyl-	993	993	0.388	96.189	1.657 ± 0.685 [c]	1.062 ± 0.173 [c]	0.888 ± 0.153 [bc]	0.074 ± 0.028 [ab]	ND
27.56	(2R,5S)-2-Methyl-5-(prop-1-en-2-yl)-2-vinyltetrahydrofuran	996	1008	12.017	86.720	ND	ND	ND	0.08 ± 0.009 [b]	0.208 ± 0.024 [c]
29.82	2,2'-Bifuran	1048	1047	-0.880	74.927	0.041 ± 0.009 [b]	ND	ND	ND	ND
	**Monoterpenes**									
24.67	Bicyclo[3.1.0]hex-2-ene, 2-methyl-5-(1-methylethyl)-	935	929	-12.639	76.357	ND	ND	0.068 ± 0.02 [b]	ND	ND
25.23	(1R)-2,6,6-Trimethylbicyclo[3.1.1]hept-2-ene	946	929	-17.394	83.565	ND	0.087 ± 0.057 [ab]	0.127 ± 0.051 [b]	0.062 ± 0.027 [ab]	ND
27.32	.beta.-Myrcene	991	991	0.348	88.359	0.159 ± 0.038 [a]	3.477 ± 0.401 [b]	7.58 ± 0.758 [c]	11.426 ± 0.53 [d]	ND
28.53	.alpha.-Phellandrene	1018	1005	-12.895	94.204	ND	0.05 ± 0.011 [ab]	0.112 ± 0.046 [bc]	0.18 ± 0.002 [c]	0.265 ± 0.038 [d]
28.98	1,3-Cyclohexadiene, 1-methyl-4-(1-methylethyl)-	1028	1017	-11.268	91.895	ND	0.108 ± 0.02 [b]	0.192 ± 0.012 [c]	0.264 ± 0.016 [d]	0.376 ± 0.029 [e]
29.32	trans-.beta.-Ocimene	1036	1049	12.995	97.828	ND	ND	1.534 ± 0.045 [b]	3.031 ± 0.193 [c]	4.116 ± 0.246 [d]
29.57	Limonene	1042	1018	-23.662	98.304	4.016 ± 0.391 [b]	ND	ND	14.957 ± 1.406 [c]	14.094 ± 0.905 [c]
29.75	.beta.-Phellandrene	1046	1031	-14.999	91.839	ND	0.194 ± 0.047 [b]	0.341 ± 0.059 [cd]	0.257 ± 0.018 [bc]	0.368 ± 0.022 [d]
29.86	.beta.-Ocimene	1048	1037	-11.402	98.471	ND	ND	2.85 ± 0.506 [b]	5.457 ± 0.761 [c]	ND
29.86	1,3,6-Octatriene, 3,7-dimethyl-, (Z)-	1048	1038	-10.463	98.579	ND	1.377 ± 0.107 [a]	ND	6.814 ± 1.1 [b]	7.892 ± 0.734 [b]
30.74	.gamma.-Terpinene	1068	1060	-8.533	76.093	ND	0.122 ± 0.056 [a]	ND	0.196 ± 0.049 [ab]	0.353 ± 0.16 [b]
31.28	cis-Linaloloxide	1081	1066	-15.017	85.859	ND	ND	ND	0.09 ± 0.012 [b]	0.162 ± 0.038 [c]
31.94	trans-Linalool oxide (furanoid)	1096	1086	-10.141	70.682	ND	ND	0.077 ± 0.016 [b]	0.164 ± 0.045 [c]	0.201 ± 0.032 [c]
32.01	Cyclohexene, 1-methyl-4-(1-methylethylidene)-	1098	1088	-9.663	94.425	ND	0.169 ± 0.035 [b]	ND	ND	ND
33.37	2,4,6-Octatriene, 2,6-dimethyl-	1131	1131	-0.008	94.481	ND	ND	0.083 ± 0.008 [a]	1.008 ± 0.078 [b]	1.367 ± 0.125 [c]
33.37	2,4,6-Octatriene, 2,6-dimethyl-, (E,Z)-	1131	1131	-0.058	97.959	ND	ND	ND	0.163 ± 0.021 [b]	0.332 ± 0.038 [c]
33.64	2,6-Dimethyl-1,3,5,7-octatetraene, E,E-	1138	1131	-6.719	87.791	ND	ND	ND	0.041 ± 0.012 [b]	0.089 ± 0.008 [c]

RT (min)	Compound name	RI	LRI	Delta RI	Match Factor	Control	C.S 2%	C.S 4%	C. L 2%	C. L 4%
33.79	(E)-4,8-Dimethylnona-1,3,7-triene	1141	1116	-25.243	85.528	ND	ND	0.066 ± 0.006 [b]	ND	ND
34.08	Limonene oxide, trans-	1149	1138	-10.570	75.321	ND	0.179 ± 0.019 [b]	0.393 ± 0.034 [c]	0.641 ± 0.031 [d]	0.885 ± 0.037 [e]
34.22	Limonene oxide	1152	1139	-12.975	89.051	ND	0.677 ± 0.085 [c]	1.631 ± 0.188 [d]	0.276 ± 0.058 [b]	0.277 ± 0.043 [b]
	**Sesquiterpenes**									
42.46	.alpha.-Cubebene	1371	1351	-19.571	83.001	ND	ND	0.072 ± 0.002 [b]	ND	ND
43.51	isoledene	1400	1375	-25.060	82.306	0.086 ± 0.043 [b]	ND	ND	ND	ND
43.51	Copaene	1406	1376	-30.043	93.467	0.181 ± 0.006 [a]	0.145 ± 0.102 [a]	0.171 ± 0.125 [a]	0.164 ± 0.015 [a]	0.148 ± 0.032 [a]
43.96	Cyclohexane, 1-ethenyl-1-methyl-2,4-bis(1-methylethenyl)-, [1S-(1.alpha.,2.beta.,4.beta.)]-	1414	1391	-22.599	88.130	0.064 ± 0.008 [b]	ND	ND	0.074 ± 0.015 [b]	0.114 ± 0.014 [c]
45.33	cis-.alpha.-Bergamotene	1455	1415	-19.773	97.747	ND	0.394 ± 0.076 [c]	1.165 ± 0.051 [d]	0.24 ± 0.008 [b]	0.435 ± 0.038 [c]
46.11	Aromandendrene	1478	1440	-38.106	95.301	0.169 ± 0.011 [b]	ND	ND	0.18 ± 0.016 [bc]	0.207 ± 0.014 [c]
46.33	Bicyclo[2.2.1]heptane, 2-methyl-3-methylene-2-(4-methyl-3-pentenyl)-, (1S-endo)-	1485	1448	-22.879	75.628	ND	ND	0.03 ± 0.004 [b]	0.14 ± 0.018 [c]	ND
47.63	.beta.-Bisabolene	1525	1509	-16.286	98.325	ND	0.122 ± 0.048 [a]	0.428 ± 0.031 [a]	2.323 ± 0.124 [b]	5.132 ± 0.747 [c]
47.78	Naphthalene, 1,2,3,5,6,7,8,8a-octahydro-1,8a-dimethyl-7-(1-methylethenyl)-, [1R-(1.alpha.,7.beta.,8a.alpha.)]-	1530	1492	-38.130	93.786	ND	0.052 ± 0.005 [b]	0.117 ± 0.007 [c]	ND	ND
48.27	Naphthalene, 1,2,3,5,6,8a-hexahydro-4,7-dimethyl-1-(1-methylethyl)-, (1S-cis)-	1546	1524	-21.924	83.038	ND	0.035 ± 0.021 [ab]	0.084 ± 0.018 [c]	0.055 ± 0.01 [bc]	0.147 ± 0.011 [d]
	**Phenols**									
51.05	2,6-Bis(1,1-dimethylethyl)-4-(1-oxopropyl)phenol	1636	1640	4.164	77.281	0.008 ± 0.001 [b]	ND	ND	0.011 ± 0.003 [b]	0.017 ± 0.004 [c]
41.20	Phenol, 4-(1-methylpropyl)-	1335	1315	-20.138	84.917	0.048 ± 0.002 [b]	0.072 ± 0.006 [c]	0.077 ± 0.01 [c]	ND	ND

Note: RI: experimental retention index; LRI: reference linear retention index; Delta RI: difference between the experimental retention index and the reference index; Match Factor: mass spectral similarity score obtained by comparison with the NIST 17 library. Compound identification was considered tentative when the Match

Factor score was greater than 70 and the RI difference was within the range of −40 to +40. RT: retention time; ND: not detected. Relative abundances are expressed as mean ± standard deviation (n=3). Different letters indicate statistically distinct groups among the types of sweet bread according to Tukey's test (sig. ≤ 0.05).
